# Social contact from birth influences personality traits of group-housed dairy calves

**DOI:** 10.3168/jdsc.2023-0383

**Published:** 2023-08-19

**Authors:** K.N. Gingerich, E.E. Lindner, S. Kalman, E.K. Miller-Cushon

**Affiliations:** Department of Animal Sciences, University of Florida, Gainesville, FL 32611

## Abstract

The objective of this study was to assess effects of prior social contact on the expression of personality traits in group-housed calves. Holstein heifer and bull calves were housed in either individual pens (n = 16), or in pairs (n = 8 pairs) at birth before they were mingled between treatments and moved to group pens at approximately 2 wk of life (8 calves/pen). During wk 4 of life, calves were tested in a series of standardized behavioral tests: an open field test, a novel object test, an unfamiliar calf test, and an unfamiliar human test. Responses in the open field test and novel object test were analyzed using principal component analysis, yielding 2 factors interpreted as bold and inactive/grooming. Calves housed in pairs before grouping had greater scores for bold and tended to have lower scores for inactive/grooming. Responses in the unfamiliar calf and human tests were similarly analyzed, yielding 3 factors interpreted as calf-directed, active, and human-directed. Calves housed in pairs before grouping had lower scores for calf-directed, tended to have lower scores for active, and scores for human-directed did not differ. Following grouping, average daily gain, milk replacer intake, and meal frequency did not differ between previous housing treatments. Our results suggest that behavioral traits reflective of personality in group-housed calves may be influenced by social contact from birth even following adaptation to group-housing.

## Highlights

• Calves were housed individually or in pairs before group-housing at 2 weeks of age.
• Previously paired calves approached a novel object more readily at 4 weeks of age.
• Social contact early in life may affect personality traits of group-housed calves.

## Graphical Abstract

**Figure:**
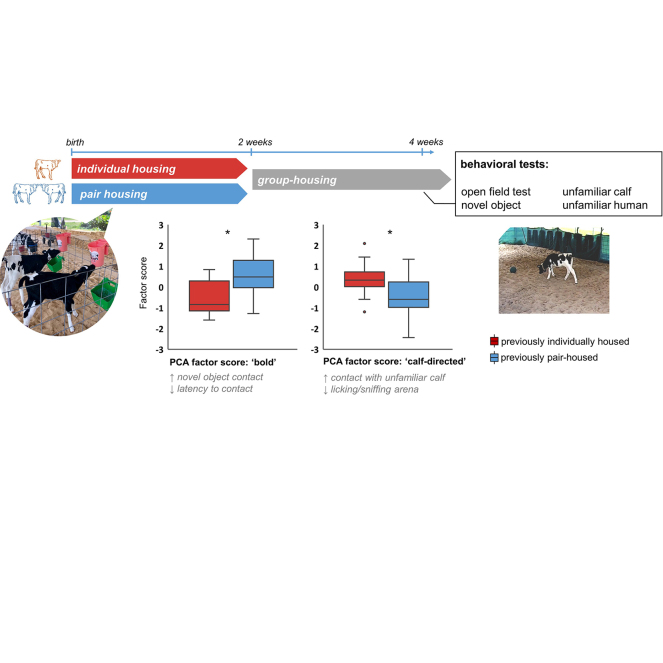
**Summary:** We characterized the effects of social contact during the first 2 weeks of life on behavioral development of dairy calves following subsequent group-housing. Behavioral responses in standardized behavioral tests indicated that calves housed in pairs before grouping approached a novel object more readily, were more active, and interacted less with an unfamiliar calf. Behavioral traits reflective of personality in group-housed calves may thus be influenced by early social contact even after adaptation to a group pen.

There is growing adoption of social housing for dairy calves, yet the age of introduction to social housing is variable, such that many calves are socially isolated for some time after birth. Evidence across species indicates that early-life social isolation has long-term adverse consequences for individual stress resilience and behavioral development (Parker and Maestripieri, 2011; Kinley et  al., 2021). For dairy calves, social housing during the preweaning period benefits cognitive development (Gaillard et al., 2014) and social bonding (Lindner et al., 2022). However, even where social housing is common, calves may be housed individually for a short time after birth (e.g., median of 2 wk in Norway; Johnsen et al., 2021). Effects of short-term social isolation on behavior of group-housed calves has not been widely studied, although some evidence suggests incremental improvements in behavioral flexibility with earlier introduction to social housing (Meagher et al., 2015).

Social environment in early life, along with other early experiences, influences the development of personality traits (Stamps and Groothuis, 2010), defined as individually variable behavioral traits that are consistent across time and context (reviewed by Kaiser and Müller, 2021). Personality is of growing interest given associations with outcomes that have implications for animal performance and welfare, including feeding behavior and weight gain in dairy calves (Neave et al., 2018) and milk production (Hedlund and Løvlie, 2015) and judgment bias (Kremer et al., 2021) in cows. While social housing throughout the preweaning period is known to reduce avoidance of novelty (objects: De Paula Vieira et al., 2012, and feeds: Whalin et al., 2018), there are gaps in our knowledge of how socialization in the first weeks of life may influence personality of group-housed calves.

The objective of this study was to assess the effect of early-life social contact, with calves reared individually or in pairs for the first 2 wk of life, on the expression of personality traits in group-housed calves. We measured responses in standardized behavioral tests commonly used to assess personality and predicted that previously pair-housed calves would be less avoidant of novelty than calves previously reared individually.

Holstein heifer and bull calves (n = 32; 16 heifers and 16 bulls) were enrolled at birth and managed according to the standard operating procedures (University of Florida Dairy Unit; Hague, FL). Procedures were approved by the University of Florida Institutional Animal Care and Use Committee (protocol no. 201909416).

Calves were alternately assigned to either individual (1.9 × 0.8 m; n = 16 calves; 8 heifers and 8 bulls) or double (1.9 × 1.6 m; n = 16 calves/8 pairs; 8 heifers and 8 bulls) wire mesh pens. Individual and pair pens were interspersed in the same barn. Sample size justification and management of calves during these first weeks of life were described previously by Lindner et al. (2021), who also described response to initial grouping. While housed in individual or pair pens, calves were provided 8 L/d of pasteurized waste milk via teat buckets in 2 daily meals. At approximately 2 wk of age (15.7 ± 2.2 d of age; mean ± SD), calves were moved to groups of 4 (age range of 4.6 ± 1.4 d; mean ± SD) composed of calves from both previous housing treatments. After 1 wk, adjacent pens were combined, forming groups of 8 (7.4 × 16.0 m), in which calves remained for the remainder of the study. Upon grouping, calves were allotted 12 L/d milk replacer (28% CP and 15% fat; Southeast Milk Inc., Mayo, FL) by an automated milk feeder (DeLaval CF1000X, DeLaval, Kansas City, MO). Calves had ad libitum access to water and pelleted calf starter (22% CP and 2% fat; Ampli-Calf Starter Warm Weather, Purina Animal Nutrition LLC, Shoreview, MN) for the entire study period. Calves were monitored by a veterinarian and no health events required exclusion from the study. Calves were weaned gradually over 10 d, beginning at 43 ± 2 d of age.

In wk 4 of life (27.2 ± 3.5 d of age) calves were exposed to a series of standardized behavioral tests used to assess individual personality traits (adapted from previously developed tests: Neave et al., 2018; Lecorps et al., 2018). All tests began at 0900 h and lasted for 10 min. Tests took place in a novel testing arena (7 by 13 m) with sand flooring and opaque walls (fence covered with a dark green tarp) and a roof. A stimulus marker was drawn 2.5 m from the start box to ensure consistent placement of novel stimuli for each test. Calves were haltered, moved into an individual mesh pen, and led to the testing arena. The test began once the halter was removed and the calf had all 4 legs in the testing arena. Personnel were partially blind to pre-grouping housing treatment (possible memory from prior calf management).

Calves were tested in a random order within pen for every test, and each calf received 4 tests (1/day) in the same order: (1) open field test in which no stimulus was placed on the marker; (2) novel object test, where a green kick ball (22 cm), was placed on the stimulus marker; (3) unfamiliar calf test, where 2 calves (chosen from calves that had been tested in the previous week) were placed in individual pens on either side of the stimulus marker, 15 cm apart; and (4) unfamiliar human test. For the unfamiliar human test, the human stood motionless on the stimulus marker for 5 min, with neutral gaze directed downward. Then the unfamiliar human slowly walked toward the calf's location (at the time of the 5 min mark) while looking toward the ground, stopped, and extended a hand. She remained in this location for the remainder of the 10-min test. The same person wearing the same clothes conducted each test.

Each test was video recorded by a camera (Hero 7 Black; GoPro Inc., San Mateo, CA) mounted 2 m from the ground with a complete view of the testing arena. Behavior was recorded continuously from video using BORIS (Friard and Gamba, 2016). We recorded the duration standing still, licking/sniffing (muzzle near the walls or floor of the arena), self-grooming (calf contacts any part of its body with its mouth, or uses back foot to contact head/neck), and activity (number of times the calf crossed into a new quadrant; test arena divided into 4 equal quadrants). For the novel object, unfamiliar calf, and unfamiliar human test, we recorded latency to contact and contact duration (muzzle within approximately 5 cm of the stimulus). For the unfamiliar human test, these behaviors were recorded during the entire 10 min test, regardless of whether the human was stationary or approaching. One observer characterized all behavior from video (intraobserver reliability was calculated for one day of video collection, with Cohen's kappa ≥0.95, as calculated in BORIS).

To describe effects of early-life social housing on performance outcomes during preweaning group-housing, calf growth and milk intake were monitored. Calves were weighed using a floor scale at birth, upon initial introduction to group-housing, at the beginning of weaning, and at the conclusion of weaning. Milk intake and meal patterns were obtained from the autofeeder, describing daily milk intake and frequency of rewarded and unrewarded visits. These data were summarized by period: preweaning (from introduction to the autofeeder at approximately 2 wk of age until weaning commenced) and weaning (10-d period).

Data were analyzed using SAS (version 9.4; SAS Inst. Inc.). All variables from behavioral tests were expressed as a percentage of test time, except for activity, which was the number of quadrants entered. Data recorded in the behavioral tests (described in Table 1) were analyzed using principal component analysis (**PCA**; using Proc Factor in SAS) to reduce correlated measures across tests and to facilitate interpretation of personality traits. Variables were divided between 2 PCA to separately focus on general response to novelty and aspects of sociality and to meet criteria for sampling adequacy. We first conducted a PCA including variables describing responses to the open field and the novel object tests (referred to as novelty PCA). Five variables were included: standing still (averaged across tests), activity (no. of quadrants entered; averaged across tests), self-grooming (averaged across tests), contact with the novel object, and latency to contact the novel object. Duration of licking/sniffing the test arena was initially included but excluded in the final analysis due to low communality estimates. Variables were screened for normality, and duration of contact with novel object and self-grooming were square-root transformed. A second PCA was conducted using tests including variables describing responses within the unfamiliar calf and unfamiliar human tests (referred to as social PCA). Seven variables were included: standing still (averaged across tests), licking/sniffing the test arena, activity (averaged across tests), contact with the unfamiliar calf, latency to contact the unfamiliar calf, contact with the unfamiliar human, and latency to contact the unfamiliar human. Self-grooming was initially included but excluded in the final analysis due to low communality. Latency to contact the novel object and unfamiliar calf were log-transformed and contact with the unfamiliar human was square-root transformed to meet assumptions of normality. Behaviors that were averaged across tests (squares entered, standing, self-grooming, licking/sniffing) were correlated (*P* < 0.1; Pearson correlation coefficients calculated with Proc CORR). For these PCA, the correlation matrix was computed, as variables were measured on different scales and had different variances. Factors were retained if eigenvalues >1 and were subjected to orthogonal (varimax) rotation. The variables included in each PCA met criteria for communality (novelty PCA >0.54 and social PCA >0.56) and Kaiser-Meyer-Olkin measure of sampling adequacy (novelty PCA = 0.56 and social PCA = 0.52). Scores for each of the PCA factors were calculated for individual calves using standardized scoring coefficients estimated using the regression method.

**Table 1:** Behavioral responses (mean and SD) of calves (n = 32) tested in open field, novel object, unfamiliar calf, and unfamiliar human tests at 27.2 ± 3.5 d of age

Behavior	Mean	SD	Range
Open field test			
Activity (no. of quadrants entered)	27.3	10.6	(9, 51)
Standing still (%)	61.9	9.8	(32.4, 81.1)
Self-grooming (%)	0.85	1.1	(0, 4.3)
Licking and sniffing (%)	31.1	14.2	(10.1, 71.0)
Novel object test			
Latency to contact object (%)	50.8	43.0	0.9–100
Duration of contact (%)	2.3	4.0	0–19.8
Activity (no. of quadrants entered)	18.7	8.3	(4, 35)
Standing still (%)	74.5	14.2	(18.7, 92.4)
Self-grooming (%)	0.62	1.1	(0, 4.8)
Licking and sniffing (%)	28.1	13.6	(1.2, 58.2)
Unfamiliar calf test			
Latency to contact unfamiliar calf (%)	7.3	9.6	0.7–52.0
Duration of calf contact (%)	26.4	11.5	1.9–49.3
Activity (no. of quadrants entered)	36.8	20.1	(8, 86)
Standing still (%)	70.1	9.8	(58.6, 84.2)
Self-grooming (%)	1.1	1.8	(0, 7.3)
Licking and sniffing (%)	20.7	12.9	(2.2, 57.9)
Unfamiliar human test			
Latency to contact unfamiliar human (%)	30.3	36.7	1.2–100
Duration of human contact (%)	14.1	23.2	0–87.3
Activity (no. of quadrants entered)	19.2	15.5	(0, 60)
Standing still (%)	75.7	17.3	(32.3, 98.1)
Self-grooming (%)	1.2	2.3	(0, 10.3)
Licking and sniffing (%)	27.6	15.5	(1.4, 60.2)

We analyzed effects of previous housing treatment on individual factor scores using general linear mixed models (Proc MIXED in SAS), with previous housing treatment, sex, and birth weight as fixed effects, group as a random effect, and calf within pre-grouping housing pen (describing the pre-grouping paired pen for pair-housed [**PH**] calves or 2 adjacent individual pens for individually housed [**IH**] calves) as a random effect, following guidelines for analyzing pen level data described by St-Pierre (2007). Model conditional residuals were screened for normality. No data were excluded from analysis. Significance was declared at *P* < 0.05, and trends were reported if 0.05 ≤ *P* ≤ 0.10.

Principal component analysis revealed factor scores reflective of different personality traits (factor loadings and the correlated personality trait interpretation for each factor are reported in Table 2). For the novelty PCA, the first factor explained 43.7% of the variance and contained a high positive loading for contact with the novel object and negative loading for latency to touch the novel object (factor interpreted as bold) and the second factor explained 28.9% of the variance and contained high positive loadings for standing still and self-grooming and a high negative loading for activity (this factor was labeled inactive/grooming). For the social PCA, the first factor explained 37.4% of the variance and contained high positive loading for contact with the unfamiliar calf and a negative loading for licking and sniffing the arena (this factor was labeled calf-directed), the second factor explained 25.8% of the variance and contained a high negative loading for standing still and positive loading for activity (this factor was labeled active), and the third factor explained 15.7% of the variance and contained a high negative loading for latency to contact the unfamiliar human and positive loading for duration of contact with the unfamiliar human (this factor was labeled human-directed).

**Table 2:** Coefficients (loadings^1^) of the eigenvalues for the first 2 factors extracted by principal component analysis (PCA) of behavioral measures in the open field and novel object tests (novelty PCA) to assess calf (n = 32) responses to novel environments, and the unfamiliar calf and unfamiliar human tests (social PCA) to assess responses to novel social environments

Variable^2^	Factor 1	Factor 2	Factor 3
Novelty PCA			
Contact with novel object	0.91*	−0.20	—
Standing still	−0.15	0.88*	—
Self-grooming	0.15	0.80*	—
Latency to contact novel object	−0.89*	0.09	—
Activity	0.41	−0.60*	—
Eigenvalues	2.18	1.45	—
Variance explained (%)	43.6	28.9	—
Interpretation	Bold	Inactive/grooming	—
Social PCA			
Standing still	0.18	0.90*	0.20
Licking/sniffing	−0.81*	0.13	−0.32
Latency to contact unfamiliar calves	−0.51	0.49	−0.25
Contact with unfamiliar calf	0.91*	0.20	0
Latency to contact unfamiliar human	−0.07	0.17	−0.89*
Contact with unfamiliar human	0.25	0.10	0.88*
Activity	0.09	−0.83*	0.20
Eigenvalues	2.61	1.80	1.10
Variance explained (%)	37.3	25.8	15.7
Interpretation	Calf-directed	Inactive	Human-directed

1 High loadings (≥0.60) are indicated with an asterisk.

2 All variables calculated as % of total test time except for activity (number of quadrants entered). Standing still, self-grooming, licking/sniffing, and activity were averaged across both tests.

Previous social housing affected 4 out of the 5 personality factors revealed in the 2 PCA (Figure 1). Calves housed in pairs rather than individually before grouping had greater bold factor scores (*F*_1,14_ = 9.39; *P* = 0.0084; Figure 1a) and tended to have lower scores for inactive/grooming (*F*_1,14_ = 3.54; *P* = 0.081; Figure 1b). From the social PCA, calves housed in pairs before grouping had lower scores for calf-directed (*F*_1,14_ = 7.86; *P* = 0.014; Figure 1c) and tended to have greater scores for inactive (*F*_1,14_ = 4.26; *P* = 0.058; Figure 1d). Scores for human-directed did not differ between previous housing treatments (0.02 vs. −0.051; PH vs. IH; SE = 0.30; *F*_1,14_ = 0.3; *P* = 0.87).

**Figure 1:**
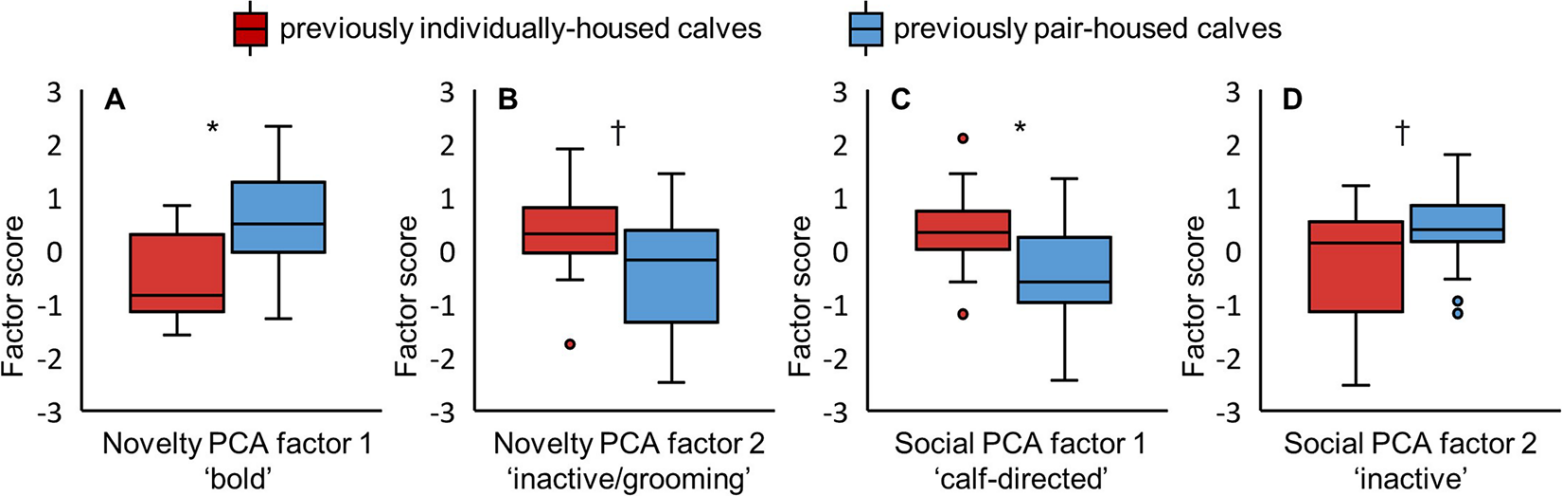
Personality factor scores from (A and B) the open field and novel object tests (novelty principal component analysis [PCA]) and (C and D) the unfamiliar human and unfamiliar calf tests (social PCA), conducted at 27.2 ± 3.5 d of age, for calves (n = 32) previously housed individually (shown in red) or in pairs (shown in blue) before grouping at 15.7 ± 2.2 d of age. Significant differences between previous social housing treatments are indicated by *P < 0.05 and †*P* < 0.1. The box extends from the 25th to the 75th percentile with a line at the median. Whiskers extend from the box a distance of 1.5 times the interquartile range with values outside this range shown as circles.

There was no effect of pre-grouping social contact on milk intake, following introduction to group-housing at approximately 2 wk of age and before weaning (8.79 vs. 8.50 L/d; IH vs. PH; SE = 0.60; *P* = 0.46) or visit frequency (3.29 vs. 3.18; IH vs. PH; SE = 0.095; *P* = 0.27) and preweaning average daily gain did not differ between treatments (1.0 vs. 1.0 kg/d; IH vs. PH; SE = 0.037; *P* = 0.93). During weaning, there was no effect of previous social housing on milk intake (*P* = 0.95) or the number of rewarded (2.16 vs. 2.21; IH vs. PH; SE = 0.24; *P* = 0.56) or unrewarded visits (17.4 vs. 19.2 visits/d; IH vs. PH; SE = 2.91; *P* = 0.39). Average daily gain during weaning did not differ between treatments (0.28 vs. 0.34 kg/d; IH vs. PH; SE = 0.11; *P* = 0.69).

In this study, we used a series of standardized behavioral tests which have previously shown responses in dairy calves which are repeatable over time (Van Reenen et al., 2005; Neave et al., 2020) to assess how early-life social contact affects the development of personality traits. We found that previously pair-housed calves had greater scores for the bold trait, describing reduced latency to contact the novel object and greater duration of contact. This is consistent with effects of social housing throughout the entire preweaning period (De Paula Vieira et al., 2012; Meagher et al., 2015). We might speculate that bolder calves may adapt more easily to housing transitions. However, we found previously that prior social housing did not affect use of novel pen resources upon introduction to group-housing, including brushes and hay (Lindner et al., 2021), which indicates a need to better understand how individual responses in behavioral tests are associated with behavior expressed in the home social environment.

We also found that calves reared individually before grouping had greater scores for the factor we interpreted as calf-directed, based on more time in contact with the unfamiliar calf and less time licking/sniffing the testing arena, suggesting an effect of early-life social contact on allocation of attention toward the unfamiliar calf versus investigating the testing arena. Effects of socialization on response to unfamiliar calves are varied; Jensen and Larsen (2014) described that calves reared socially (group pens or physical contact) spent more time sniffing an unfamiliar calf than calves reared with only visual or auditory contact with other calves. However, similar to the present findings, De Paula Vieira et al. (2012) found that individually housed calves spent more time interacting with an unfamiliar calf and were generally more reactive, defecating more frequently while exploring the testing arena less. In the present study, differences in this factor may also be explained by a greater motivation in previously pair-housed calves for investigating the testing arena (licking/sniffing parts of the arena) rather than avoidance of the unfamiliar calf per se.

Previously pair-housed calves tended to have lower scores describing inactive/grooming in the open field and novel object tests. Increased self-grooming has been previously reported to coincide with inactivity in behavioral tests in calves (Bučková et al., 2021) and cows (Herskin et al., 2004), and was interpreted as a potential displacement behavior, indicative of increased fear. In comparison, in the unfamiliar calf and human tests, we found that calves previously housed in pairs were more inactive than individually housed calves, which may reflect increased habituation to the testing arena since these 2 tests were conducted second. Previous findings suggest that socially reared calves showed declining exploration of a novel object more rapidly than individually housed calves (Gaillard et al., 2014). We found no effects of previous housing treatment on the factor described as human-directed. Previously, socially housed calves have been found to interact less with a human during approach tests (Lensink et al., 2001; Doyle et al., 2022). However, Doyle et al. (2022) found that differences in human-directed behavior were more minimal in approach tests conducted in a testing arena compared with the home pen. Our findings suggest that previous social contact before group-housing has minimal effect on human-directed behavior, although this finding may depend on the degree of human contact experienced by group-housed calves.

We found no effect of previous social contact on milk intake, consistent with previous studies examining effects of social housing during the preweaning period (Costa et al., 2015; Miller-Cushon and DeVries, 2016). However, social housing encourages increased solid feed intake later in the preweaning period, often translating to increased weight gain during the weaning period (Costa et al., 2015; Miller-Cushon and DeVries, 2016). While calves begin to nibble and investigate solid feed during the first weeks of life, starter intake remains low during this time frame and social contact for the first weeks of life may not stimulate increased starter intake (Mahendran et al., 2021). While we were not able to measure solid feed intake in the present trial, weight gain during weaning was not affected by degree of social contact before group-housing.

In summary, we found that calves housed in pairs before grouping were more bold, characterized based on latency and duration of contact with a novel object, compared with calves housed individually before grouping. We also found differences in response to an unfamiliar calf and activity in the testing arena, with previously pair-housed calves showing more activity during initial exposure to the arena and less activity during later tests. These results suggest that early-life social contact has potential to influence personality traits in group-housed calves, and further work is needed to understand the longevity of these effects and implications for management and animal welfare.
